# The Good Enough Parenting early intervention schema therapy based program: Participant experience

**DOI:** 10.1371/journal.pone.0243508

**Published:** 2021-01-22

**Authors:** John Philip Louis, Vida Ortiz, Joanna Barlas, Joyce Sue Lee, George Lockwood, Wayne Freeman Chong, Karen McDonald Louis, Patricia Sim

**Affiliations:** 1 Persatuan Kebajikan HOPE *Worldwide* Kuala Lumpur, Kuala Lumpur, Malaysia; 2 HOPE *Worldwide* Singapore, Singapore, Singapore; 3 James Cook University, Singapore, Singapore; 4 Schema Therapy Institute Midwest, Kalamazoo, Michigan, United States of America; South African Medical Research Council, SOUTH AFRICA

## Abstract

**Background and objectives:**

Schema therapy (ST) has become a mainstream therapy for the treatment of psychopathology and has been validated through a series of large scale, international randomized control trials. Among other things, schema therapy emphasizes the meeting of core emotional needs in children by primary caregivers as these unmet needs continue to adversely affect their lives into adulthood. An early intervention parenting program has been developed to help parents meet these core emotional needs in order to prevent the development of psychopathology in the first place. The program, Good Enough Parenting, is equally focused on reducing problems and strengthening parenting practices, regardless of where the child is on the “disordered to well-being continuum”. This study aims to explore “patient experience” by users of this program. Best clinical research guidelines advocate that participants should be used as collaborators rather than pure recipients; this process should predate large scale trials.

**Design:**

An exploratory qualitative study with 55 parent-participants of Good Enough Parenting was conducted.

**Methods:**

One-to-one interviews were conducted with participants, using critical incident technique and guided by semi-structured interview schedule, to explore their experiences with the program. Transcripts were then analyzed using thematic analysis.

**Results:**

Coding showed a high degree of inter-rater reliability (kappa value of 0.78). The themes that emerged were Cultivating Awareness of Parents’ Own Schemas, Cultivating Intentionality, Working through Developmental Issues, Responses to Challenges at Home, Performing Multiple Roles, and the Learning Process. Participants overwhelmingly reported satisfaction within these key themes.

**Conclusions:**

The results support the development of the program and the choice of “participant reported outcome measures” for use in subsequent randomized controlled trials.

## Introduction

Children who experience adverse childhood experiences are at greater risk of mental health problems in adulthood [1–3]. Early intervention programs that involve parents are an effective [4] and economically viable [5] way to mitigate this risk. Schema therapy is an integrative theory and treatment that combines elements of cognitive behavioral, emotion-focused, attachment and psychodynamic models. Schema therapy has considerable empirical support [6] for the underlying model and its efficacy in treating adults with disorders ranging from depression [7] to personality disorders [8, 9], including those with borderline features [10–12]. An early intervention schema therapy-based parenting program has been authored and designed, known as Good Enough Parenting (GEP) [13, 14]. It is aimed at the prevention of psychopathology before it develops, as well as the promotion of resilience and healthy functioning.

Good Enough Parenting has been increasingly and widely implemented since its development in 2009 [14]. It is rooted in the empirically supported schema therapy model which forms the basis of the well validated schema therapy interventions. Good Enough Parenting is referenced in several recent articles exploring key constructs in schema therapy [15–17]; there is a professionally developed program to train facilitators and trainers [14], and there is a book written for the general public describing the program [13]. Increasingly, Good Enough Parenting is being accepted and used internationally based on the perceived fit with stakeholder needs and its apparent popularity amongst participants. It is being used in Malaysia, Singapore, India, Indonesia, Cambodia, Vietnam, Thailand, Japan, Lebanon, Ukraine, Russia, Norway, Sweden, France, the United States, and Australia, as well as parts of South America and Africa.

Since the Good Enough Parenting program has gained acceptance in different cultures, it is important to provide an overview of its link to current parenting literature. The program is built on schema theory literature [6] to consider a broader range of adaptive and maladaptive parenting patterns that relate closely to the core emotional needs of children [15, 16, 18], and thereby prevent the development of psychopathology. It is also important to see if these types of parenting patterns cut across cultures, and to identify key patterns, if any, that are culture specific. As such, consideration will also be given to the measures underpinning Good Enough Parenting [15, 16] and their links to other established instruments measuring parenting patterns.

Interest in parenting constructs and their effect on developmental outcomes in children began about 75 years ago with the identification of two parenting dimensions: autocratic and democratic [19]. This was initially expanded to three parenting dimensions based on variations in warmth and control: Authoritative (high warmth-high control), Authoritarian (low warmth-high control), and Indulgent / Permissive (high warmth-low control) [20] with a fourth dimension of Neglectful (low warmth-low control) being added later [21].

The benefits of the Authoritative parenting style have been replicated across many European and American populations but its benefits in other cultures have been questioned. For example, Chao [22] contended that the three parenting styles may have different meanings in the Chinese culture. However, subsequent research on the Authoritative parenting demonstrated its benefits in a broad range of cultures including China, Pakistan, Hong Kong, Scotland, Australia, and Argentina [23–27].

In the USA, a major study of 10,000 children clustered in 16 ecological niches showed that homes with Authoritative parenting benefitted all these groups with respect to psychosocial development, symptoms of internalized distress, and problem behavior in children. However, the influence of culture also contributed to the outcome, in that African American adolescents, and to a lesser extent, Asian American adolescents, were not as negatively affected by Authoritarian parenting as those from the White population [26]. The effect of parenting styles on different cultures has also emerged from a meta analyses of 428 studies in Western countries conducted by Pinquart and Kauser [28]. On the whole they found that while different regions and various ethnicities exhibited more similarities than differences, specific parenting styles also varied across different cultures. For example, associations of Authoritative parenting with academic achievement were stronger in non-Hispanic, White families than in Asian minorities, but associations of Authoritarian parenting with academic achievement were less negative in Hispanic families than in non-Hispanic, White families. The effects of culture and ethnicity on the outcome of parenting is not new as Darling and Steinberg [29] have noted that Authoritarian parenting was associated with fearful and timid behavior among European-American children but was conversely associated with assertiveness among African-American girls. On the other hand, Authoritative parenting was most strongly associated with academic achievement among European-American adolescents but least among Asian-African American youths. This may be due to other processes operating within a specific culture such as methods parents used to achieve the same goals [29]. The less negative effect of Authoritarian parenting also emerged in a study by Dwairy and Achoui [30] where Authoritarian parenting was not found to be associated with any negative outcomes in relation to mental health such as anxiety and depression. One study by Dwairy, Achoui, Abouserie, Farah, Sakhleh, Fayad, and Khan [31] found that the three parenting styles did not emerge distinctly but as hybrids of the three parenting styles from Baumrind’s model [20], namely Inconsistent (Permissive and Authoritarian), Controlling (Authoritarian and Authoritative), and Flexible (Authoritative and Permissive).

Positive effects of Indulgent or Permissive parenting were found in studies conducted in Sweden, United Kingdom, Spain, Portugal, Slovenia, and the Czech Republic [32], Portugal and Brazil [33] and Germany and the United States [34]. A recent study in Spain also showed that Indulgent and Authoritative parenting styles were associated with better outcomes with respect to children being less aggressive, having greater self-esteem, and greater psychological adjustments than either the Authoritarian or Neglectful parenting style [35]. This study highlighted that the Indulgent parenting style was associated with the best outcomes and was shown to be associated with personal adjustment [36] and protection against alcohol [37]. Homes with Indulgent and Authoritative parenting styles also resulted in adolescents with higher self-esteem and greater internalization of environmental values than their counterparts [38]. Together, these results suggest that “parents across the globe could be recommended to behave Authoritatively, although Authoritarian and Indulgent parenting is, to some extent, tolerable in a few cultural contexts” [28, p.75].

However, while the influence of culture may explain why Indulgent parenting style is the most optimal in some countries, such variations may also be due to the different definitions associated with the Authoritative parenting construct. For example, Garcia and Serra [39] based their definition of Authoritative parenting on Baumrind’s model [20] which was made up of two dimensions–high warmth and high control. Using the parental control scale (PCS; 4-point Likert scale from “1 = almost never true” to “4 = almost always true”), examples of items representing control were, “They make sure I know exactly what I can and cannot do”; “and “They believe in having a lot of rules and sticking to them” [39]. Such items representing control portray a very directive, over-controlling approach when higher scores on the Likert scale such as 3 or 4 were selected. However, when another measure known as the parenting style index (PSI; 4-point Likert scale, from “1 = Strongly agree” to “4 = Strongly disagree”) was used [26], the items describing control were not as dictatorial as they were in the PCS. For example, in the PSI there are three dimensions to Authoritative Parenting, namely Firm Control (e.g. “How much do your parents try to know where you go at night?”); Acceptance/Involvement (e.g. “I can count on (them) to help me out if I have some kind of problem”); Psychological Autonomy (e.g. reversed scored, “How often do your parents tell you that their ideas are correct and that you should not question them?”) [26]. Therefore, the exact definition of strictness that made up part of the Authoritative parenting construct differed depending on whether the PCS or the PSI was used. Given that different definitions of control were used, it is not surprising that different results were obtained on the efficacy of Authoritative parenting. In fact, Osorio and Gonzalez [40] investigated this very issue by using the PSI in a sample of adolescents in Spain. Results showed that the Authoritative parenting style yielded the best outcome which was consistent with findings from other Anglophone countries. The different definition of control may likely explain some of the different results regarding the optimality of the Indulgent parenting style in studies done in Spain in comparison to other Anglophone countries [40, 41]. On balance, after extensive research over the last 50 years, notwithstanding the effects of culture and ethnicity on parenting styles, the Authoritative parenting style has been shown to provide the greatest benefits to children across cultures [42].

Even though Baumrind’s [20] parenting constructs have been found to be associated with a range of developmental outcomes in children [43], there were concerns that the model was predicated on normal variations in parenting rather than deviant ones [44, 45], and therefore there was a need to look at parenting dimensions that went beyond the dimensions of control and warmth found within this cultural paradigm [28, 46, 47]. For example, other dimensions have been identified from studies showing their link to the development of Borderline Personality Disorder [48–52]. These deviant parenting patterns include invalidation of children’s emotions, various forms of abuse, neglect and overprotection.

The current and most widely used parenting instruments measuring past parenting patterns are also limited in their scope. For example, the s-EMBU (Swedish acronym for “My memories of upbringing”) has three subscales with two being maladaptive and one adaptive: Parental Rejection, Emotional Warmth, and Overprotection [53]. The Childhood Trauma Questionnaire has five maladaptive subscales [54] and no adaptive subscales. The Parental Acceptance-Rejection Questionnaire Adult version has one subscale representing Acceptance called Warmth, and three maladaptive ones representing Rejection called Hostility, Indifferent and Undifferentiated [55]. The Parental Bonding Instrument (PBI) has three parenting constructs, one adaptive subscale called Care, and the other two maladaptive ones called Overprotection and Authoritarianism [56]. The Alabama Parenting Questionnaire [57] has two adaptive constructs–Involvement, and Positive Parenting, and three other constructs involving Control called Poor Monitoring, Inconsistent Discipline and Corporal Punishment. In summary, all these instruments measuring parenting patterns consist of only three to four maladaptive constructs, and one to two adaptive ones. It may be that these parenting constructs, like Baumrind’s, though used in multicultural settings, were generally based on *normal variations* of parenting, not deviant ones. The need to relook at parenting dimensions that go beyond control and warmth as conceptualized by Baumrind [58] and its limited scope was highlighted by several important studies [28, 46, 47, 59]. Further, while the Baumrind [58] model has been used extensively in many parts of the world, studies especially in non-Western populations, have also shown that the Baumrind’s model [20] did not fit exclusively [31, 60] Given this limitation, a more nuanced parenting model is needed [47].

From the vantage point of the prevailing parenting constructs, The Good Enough Parenting program uses a broader and more nuanced range of parenting constructs that are grounded in schema theory; a theory that has clinical origins, and therefore a better grasp of maladaptive and adaptive patterns. These constructs have been found to be robust in both Eastern and Western samples, as well as in developing and developed countries [15, 16, 18]. There are seven adaptive parenting patterns known as nurturing interactions and labelled as: Autonomy Granting, Autonomy Support, Confidence and Competence, Dependability, Emotional Nurturance and Unconditional Love, Intrinsic Worth, Playfulness and Emotional Openness [15]. These seven subscales go beyond the dimensions of high warmth and high control (Authoritative) that previous models were based on [20, 21] and include dimensions of parenting such as believing in a child, providing age appropriate autonomy, cultivating intrinsic values, being dependable, providing emotional openness, and being playful. The parenting program also utilizes ten maladaptive parenting patterns known as exasperation interactions and labelled as: Competitiveness and Status Seeking, Over-Control, Disconnection and Rejection, Neglect and Insufficient Guidance, Emotional Inhibition and Deprivation, Intrusiveness and Exploitation, Overprotection and Overindulgence, Punitiveness and Abuse, Social Exclusion, Undependability and Irresponsibility [16, 18].

Both the nurturing and exasperation interactions have been developed into self-report scales. These are administered to adults when parental socialization is over and are therefore worded in the past tense to measure perceptions of past parenting experiences. These represent a fuller spectrum of maladaptive and deviant parenting patterns that go beyond the dimensions of control and warmth, represented by Authoritarian, Neglectful, and Permissive parenting styles. As a result, and not surprisingly, the nurturing interaction scale and the exasperation interaction scale demonstrated incremental validity [61]. This means that they added additional statistically significant variance in measures of well and ill being (depression, anxiety, stress, personality dispositions, and psychological well-being) over and above that contributed by several of the aforesaid established parenting scales; namely the s-EMBU, the Childhood Trauma Questionnaire, and Parental Acceptance-Rejection Questionnaire [15, 16]. Therefore, this provided justification for their development in that they were shown to not be just proxies for the existing parenting scales [61].

The nurturing interaction subscales of the Positive Parenting Schema Inventory (PPSI) [15] also correlated with subscales of the s-EMBU, PARQ and CTQ [15]. The average statistically significant correlation values of the PPSI scale with the s-EMBU, CTQ and PARQ were moderate; |r| = .40, .31 and .47 respectively. As expected, the Emotionally Nurturing and Unconditional Love subscale of the PPSI correlated positively with the Emotional Warmth scale of the s-EMBU and negatively with the Warmth scale of the PARQ (reversed scored). The PPSI subscale Autonomy Support, measuring the dimension of parents’ “belief” in the child, had the highest negative correlations (moderate in strength) with subscales of Rejection and Warmth (reversed scored) of the PARQ and Emotional Neglect of the CTQ. It also correlated positively with the Warmth scale of the s-EMBU. The PPSI (Fathers) subscale Autonomy Granting had the highest negative correlations (moderate in strength) with the Overprotection subscale of the s-EMBU [15].

For the exasperation interactions subscales, the average statistically significant correlation values with the s-EMBU, CTQ and PARQ were .30, .29, and .42, respectively. The exasperation interaction subscale Degradation and Rejection correlated the highest (moderate in strength) with the Rejection subscale of the s-EMBU. All subscales of the CTQ contained facets of Abuse and Neglect, while all the PARQ subscales contained facets of Acceptance-Rejection constructs. As expected, their highest correlation (moderate in strength) was with the Degradation and Rejection and Punitiveness subscales. The exasperation interactions subscale of Emotional Inhibition and Deprivation correlated the highest with the Warmth (negative direction) sub scale of the s-EMBU, the Emotional Abuse and Emotional Neglect sub scales of the CTQ, and the Warmth and Indifference / Neglect (score reversed) subscales of the PARQ. The Over-Control exasperation interaction sub scale correlated mostly with the s-EMBU subscales of Rejection, and Overprotection [16].

Since both these broader and more nuanced adaptive and maladaptive parenting patterns were also found to be associated with Early Adaptive Schemas and Early Maladaptive Schemas respectively [15, 16], leveraging them will help parents to meet children’s core emotional needs more effectively and thereby prevent the development of psychopathology later in life [6, 18].

### Schema therapy and Good Enough Parenting

Early maladaptive schemas [6] and early adaptive schemas [17, 62] are key constructs within the schema therapy model and the Good Enough Parenting program. At a cognitive level, schemas are the structures in which data from experiences are stored in autobiographical memory from early childhood onwards [63]. In psychotherapy broadly, schemas are thought of as the lens or filters through which people view the world, their relationships with other people, and themselves [64]. Schemas feature in traditional cognitive models where they are defined as “stable cognitive patterns” that provide rules for the processing of information and subsequent behavior [65]. Schema therapy, being an integrative approach, defines schemas more broadly including patterned affect, neurobiological reactions, and implicit and explicit memory in addition to cognitions or “core beliefs”. Schema therapy focuses on 18 early maladaptive schemas and their importance in psychopathology. These have received extensive empirical support across different populations and cultures [66–69]. More recently, 14 early adaptive schemas have been identified which are believed to promote well-being and resilience against mental disorder [17]. To date, schema therapy has yet to formally incorporate the 14 early adaptive schemas in its treatment protocol [17]. The Good Enough Parenting early intervention program is the first schema therapy-based treatment to do so, including a dual focus on the prevention of early maladaptive schemas and the enhancement of early adaptive schemas via parent training [13].

A central assumption of schema therapy is that early maladaptive schemas develop when a child’s core emotional needs are not met and that early adaptive schemas develop when they are [6, 18]. This is believed to occur in the context of parenting and to involve an interaction between child and parent and to be influenced by the temperament of each. When early maladaptive schemas are triggered in adulthood, they lead to emotional distress and problems in interpersonal relationships both directly and indirectly via maladaptive coping behaviors [6]. In contrast, when early adaptive schemas are triggered they are believed to lead to adaptive functioning that contributes to overall well-being [17]. These are believed to be related to but distinct from the parent’s own early adaptive schemas and early maladaptive schemas, although there is also evidence that parental schemas transfer to their children [70].

Core emotional needs, previously defined by Young et al. [6] and Lockwood and Perris [62], have most recently been investigated and conceptualized by Bach, Lockwood and Young [71] in relation to early maladaptive schemas, and by Louis, Davidson, Lockwood and Wood [72] in relation to early adaptive schemas. Consistent empirical support has been found for four categories of early maladaptive schemas which are believed to arise from the frustration of core emotional needs associated with maladaptive parental styles [71]. These domains are: Disconnection and Rejection, Impaired Autonomy and Performance, Impaired Limits, and Excessive Responsibility and Standards. Similarly, four categories of early adaptive schemas have been identified that are believed to arise when core emotional needs are met through patterns of positive parenting [72]. These are: Connection and Acceptance, Healthy Autonomy and Performance, Reasonable Limits, and Realistic Standards and Reciprocity. The four clusters of early maladaptive and adaptive schemas appear to run in parallel, supporting the theoretical basis of both schema therapy and the Good Enough Parenting early intervention program in terms of the relationships between core emotional needs, parenting patterns, and schemas. These four categories of early maladaptive and adaptive schemas represent unmet and fulfilled needs, respectively, by early primary caregivers. However, these four categories are not the parenting patterns themselves. The parenting patterns that are believed to fulfill or thwart core emotional needs are the nurturing interactions and exasperation interactions respectively [6, 13].

Good Enough Parenting involves parents and their children in the process of promoting the conditions needed for core emotional needs to be met, aided by specific parenting patterns [15, 16] with the goal of reducing the development of early maladaptive schemas and promoting the formation of early adaptive schemas during the formative years, which can help reach parenting socialization goals.

Parenting socialization is an adult initiated process through which the young person acquires his / her culture as well as habits and values congruent with the adaptation to that culture so that children may become responsible members of their society [73]. For many societies, parenting socialization is over when children become adults. For many cultures, the age when this transition takes place is somewhere the ages of 18 and 21, despite a new body of emerging evidence showing that the period of adolescence does not end until the age of 25 [74].

Schema theory explains that problematic adulthood behavior and dispositions as being due to a thwarting of core emotional needs that leads to the development of maladaptive schemas. These schemas, along with their associated copying styles, become pathological in that they become rigid ways of viewing and acting in relation to the world, others and oneself that are not amenable to later environmental changes or disconfirming evidence. When this occurs, the goals of parenting socialization are not reached as schemas can lead to problematic behavior and dispositions causing harm to a person’s relationships all the way into adulthood and old age. In schema therapy, an adult person with strong, active maladaptive schemas will undergo a process of “resocialization” with the therapist serving as a transitional parent figure through a process called “limited reparenting” [6]. This underscores the importance of parenting as it has lifelong effects regardless of whether the child has reached an “adult” age.

The Good Enough Parenting program takes an early intervention perspective whereby it is easier to prevent the development of maladaptive schemas than it is to change them later [6], and meeting core emotional needs adequately during childhood will help achieve parenting socialization goals. In Good Enough Parenting, parents are provided with concrete strategies and interventions to increase the number and quality of positive interactions as well as taught to recognize the presence of negative parenting patterns. They also learn how to avoid them or withdraw from them once they start [13] and to practice positive parenting patterns.

As the popularity of the program increases, there is a clear need for independent testing of the program rather than just drawing inferences from the well-supported theoretical basis and the wide support for schema therapy in adults. Guidelines for the refinement and testing of new complex interventions stress a multi-stage process in which a focus on end-user acceptability and ease of implementation are as important as showing that the intervention works in controlled conditions [75, 76]. Judging the acceptability of an intervention is partially based on understanding the experience of program participants, their views on the coherence and effectiveness of the program, and their sense of self-efficacy in implementing program techniques [77]. Participant views are important for informing the perspective of key stakeholders and the real-world success of a program [78]. Even where different interventions have been shown to be equally as effective in clinical outcomes in randomized controlled trials (RCTs), how effective they are in uncontrolled settings depends on factors such as disengagement and drop out that arise from patient experience [79]. Finally, participants’ subjective views of the legitimacy of the program and associated expectancies of change are themselves key factors in determining objective therapeutic change [80]. For these reasons, we conducted a qualitative study focusing on Good Enough Parenting participant experience as part of an evaluation program which will also feature RCTs when that step of the complex intervention framework is reached.

## Method

### Participants

The host organization and the stakeholder of this research is a global affiliate of a nongovernmental organization (NGO), an international faith-based charity headquartered in the United States, and registered as a charity in Singapore. Ethical considerations were in accordance with standards advocated by the British Psychological Society as well as the American Psychological Association. Approval to conduct this study was granted by an ethics committee consisting of a representative member of the Board of the Singapore NGO, and the NGO’s family life educators and resource speakers. The committee attests regarding voluntary participation with informed consent, no participation from vulnerable groups, no exclusion because of race, colour, religion, or marital status, participants’ anonymity, data confidentiality, no harm will be experienced by any participant, and that no incentives were given. It also confirms the exclusion of program authors in data collection and analysis and that an independent expert was involved to ensure objectivity and integrity of the process carried out.

Parents in Singapore who attended at least one Good Enough Parenting workshop session from 2009 and 2019 were contacted by the NGO over the phone and via email, explaining the purpose and method of this study. No participants were excluded because of race, color, religion or current marital status. Only those without adequate command of the English language were excluded. Participation was voluntary and consent forms were signed by each participant. 68 parents from nonclinical English-speaking community indicated interest to attend the program, but 55 were shortlisted to represent the Singapore population demographics for this exploratory qualitative study. The sociodemographic profile of the participants is presented in the results section.

### Program modules and feedback from parents and facilitators

Good Enough Parenting targets parents with children from infancy to late adolescents / young adulthood. Practical points are given which are age-specific in order to meet parenting needs at different ages. Good Enough Parenting is taught either as stand-alone modules or as full program. Module 1 is titled “Avoiding Exasperation Interactions” which provides an overview of the Good Enough Parenting model, core emotional needs, and the detailed explanations of exasperation interactions and nurturing interactions. Modules 2–5 present an in-depth discussion of each core emotional need (i.e., Connection and Acceptance; Healthy Autonomy and Performance, Reasonable Limits, and Realistic Standards and Reciprocity) and the parenting strategies that can be used in order to meet each need. Module 6 discusses Intrinsic Values and Community, as well as Repair and Reconnect, or resolving conflicts through the power of vulnerability and forgiveness.

To ensure that the program is delivered based on its intended outcomes, Good Enough Parenting is taught by the program authors and developers, along with facilitators and trainers who have completed advanced level trainings. Most of these facilitators have relevant training in counselling, social work, psychology or family life education.

Good Enough Parenting has been favorably received by parents and facilitators. Based on feedback from 645 parents in Singapore who attended the workshops from 2016 to 2019, the mean score for overall satisfaction of the program was 4.52 using a 5-point Likert scale, or 90.4%. Further, 96% of 150 facilitators who attended advanced trainings from 2017 to 2019 rated “strongly agree” and “agree” when asked to evaluate the overall course content and structure.

### Data collection

Participants were asked to reflect on their experience of parenting prior to and after utilizing the principles of Good Enough Parenting. They were interviewed using a semi-structured interview schedule which incorporated the Critical Incident Technique from May to July 2019 (see S1 Appendix).

CIT was first developed by Flanagan [81] to collect direct observations of behaviors in order to examine the effectiveness of a pilot’s performance. CIT has been used in the field of counselling and psychology [81, 82] to complement the attributes of qualitative research, including recording complex, rich data, so as to understand and capture the meaning, experience and actions of individuals as they go through their lived realities [83].

Inherent within CIT are five steps: (i) identifying general aims, (ii) planning, (iii) collecting data, (iv) analyzing data and (v) interpreting and reporting results. Table 1 highlights each step of the CIT process [81–83].

**Table 1:** Five steps in critical incident technique.

i. Identifying general aims
• Identify the research question at hand
ii. Planning
• The situations to be observed
• The observers to be familiar with topic
• How data is to be collected
• How data is to be analyzed
• Requirements of Institutional Review Board to be addressed
iii. Collecting data
• Timing of recording and reporting
• Format of reports (e.g. individual or group interviews, questionnaire)
• Size of sample
iv. Analyzing data
• Selecting a frame of reference that is related to how data is to be used
• Developing an area of major or sub-area category
• Placing incidents into categories as they are reviewed, or making simple count
v. Interpreting and reporting results
• Review for potential bias
• Limitations of research
• Transparency of data collection and analysis methods

Adapted from “The critical incident technique: A useful tool for conducting qualitative research,” by K. FitzGerald, N. S. Seale, C. A. Kerins, and R. McElvaney, 2008, Journal of Dental Education, 72(3), p. 299–304. Copyright 2008 by the American Dental Education Association.

The collection of data for CIT typically requires the respondents to share an experience. In this study, participants were asked to share incidents that happened between them and their child(ren), and to reflect on factors that contributed to the positivity or negativity of these incidents. These incidents deliberately did not require Good Enough Parenting to be considered to allow participants to talk freely about any aspects of, or approaches to, parenting. The rest of the semi-structured interview schedule was focused specifically on eliciting participants’ key learnings from Good Enough Parenting workshops such as its impact on their relationship with their child, challenges they faced, and their understanding and use of Good Enough Parenting principles. Example questions were: “What were some of the most natural, and therefore, easiest principles to apply for you?” “Looking back, how do you think you have grown as a parent?” “What nurturing interactions would you like to see more of?” What do you think are challenges that prevent nurturing interactions from happening more?” Whilst a standard interview schedule was provided, interviewers were encouraged to ask other types of second questions, like probing questions (“Could you tell me more about that?”) and interpreting questions (“Is it correct to say you feel…?”).

CIT comes with multiple advantages [84] such as documenting data from the respondents’ perspective, according to their own words, as well as providing respondents with the autonomy to decide on the incident which is most relevant to the research being conducted. This approach is inductive in nature [85] and effective when the research topic is sparsely documented [86] and useful when attempting to gather a more thorough understanding of the phenomenon [87]. Unlike other research methods, CIT does not require a fixed hypothesis, and flexibility is provided for the researcher to generate concepts about the theories, rich data consisting of a sequence of events [86, 88] and analyzing respondents’ perceptions of different cultural backgrounds [89, 90]. As this study was the first that collects data from Good Enough Parenting participants, the ability of CIT to facilitate the collection of rich and exploratory data was deemed useful.

### Development of codes and pilot testing

The first and sixth co-authors, as developers of Good Enough Parenting, were not involved in either interviewing or data analysis to avoid any unintentional bias in data collection and interpretation. The development of the codes was done over two rounds from both deductive and inductive perspectives. The first round involved developing key themes from the Good Enough Parenting book, providing a framework for the codebook. In the second round, two pilot interviews were conducted, transcribed and four new codes were developed (Context, Learning, Support Factors and Aspirations). The eventual codes which were most salient that emerged were: Principles and Interactions at Work, Context, Learning, Support Factors, Aspirations, Challenges, and Seeing Less Of. Open coding was used for the sub-node. For example, the Principles and Interactions at Work code captured all mentions of the principles that participants associate with Good Enough Parenting as well as interactions with their children since learning Good Enough Parenting. Sub nodes under this code included “good enough” and “reasonable limits”. Support factors coded for when participants recognized things that supported them in their parenting, including but not limited to practicing Good Enough Parenting. Sub-nodes included things like grandparents and community.

### Data analysis

Six transcripts were selected to be coded independently by two coders using the developed code book. The coders came together to discuss their coding twice to resolve discrepancies and check that the codebook worked and their understandings were correct. The final overall kappa coefficient for the coding is 0.78 which reflects reasonably consistent and substantial inter-coder reliability in terms of coding of the transcripts [91]. Once this was achieved, the coders applied the coding schema to the remaining transcripts. The final codes were then synthesized into a master analysis file and analyzed using thematic analysis.

Thematic analysis is one of the most common approaches to analyzing qualitative data. It is especially useful when applied to interview transcripts, as it involves examining repeating patterns or key themes that appear in the transcripts. Following Braun & Clarke’s [92] six steps of thematic analysis, codes were used to generate a set of initial themes. These were then reviewed to identify areas where they may overlap, and to check that the themes accurately represented the data they were trying to describe. The list of themes was refined; they were given titles and then written up. The following section describes each theme that emerged from the analysis.

## Results

### Sociodemographic profiles of the participants

While the ethnic distribution of Singapore’s population is 74.2% Chinese, 13.2% Malays, 9.2% Indians, and 3.4% from other minority groups [93], the sample distribution in this study was 67% Chinese, 9% Malays, 15% Indians, and 9% from other minority groups. The percentage of male and female participants were 51% and 49% respectively. The mean age of the participants was 46.55 years, and the Standard Deviation was 7.32 years. As for marital status, 96% classified themselves as married and 4% as divorced.

98% of the participants attended the full program of at least 21 hours, or 3 full days, conducted by the program authors and developers; the remaining 2% attended at least one module. 27% of the participants attended Good Enough Parenting for the first time from 2009–2010; 60% from 2011–2015; and 13% from 2016–2019.

93% of the participants attended the workshop more than once, providing a refresher to the parents as their children grew up and as the number of their children increased. These workshops were taught either by the authors and developers or other accredited trainers. The parents learned age-specific practical applications, along with updates on key concepts such as exasperation interactions, nurturing interactions, and positive schemas. The remaining 7% attended once and less than one year since the time of the actual interview. Since Good Enough Parenting underscores the importance of community support, 91% of the participants attended at least one support group or were still actively part of parent support groups once every two months, or six times a year, which reinforced the teachings of Good Enough Parenting.

As for the number of children, 14% of the participants have only one child; 40% have two children; 42% have three children; and 4% have four children. The distribution of the ages of the children whose parents participated in the study were as follows: 36% (0 to 10 years), 40% (11 to 19 years), 22% (20 years or above), and 2% were not disclosed. As to the age of the participants’ first child since learning Good Enough Parenting, 60% were from 0–10 years; 38% were from 11–19 years; and only 2% with first child aged over 20 years. (See Table 2).

**Table 2:** Sociodemographic characteristics of the community sample in Singapore (n = 55).

Characteristics	Categories	*n* (%)
Gender	Men	28 (51%)
	Women	27 (49%)
Age (years)	30–39	11 (20%)
	40–49	22 (40%)
	> = 50	22 (40%)
Ethnicity	Chinese	37 (67%)
	Malay	5 (9%)
	Indian	8 (15%)
	Others	5 (9%)
Marital Status	Married	53 (96%)
	Divorced	2 (4%)
Completion of GEP program	Completed all modules	54 (98%)
	Attended at least 1 module	1 (2%)
Familiarity on GEP program	Attended more than once	51 (93%)
	Attended once	4 (7%)
Year attended first workshop	2009–2010	15 (27%)
	2011–2015	33 (60%)
	2016–2019	7 (13%)
Participation in GEP Support Group	Yes	50 (91%)
	No	5 (9%)
Number of Children	1 child	8 (14%)
	2 children	22 (40%)
	3 children	23 (42%)
	4 chidren	2 (4%)
Ages of parents’ children In the sample	0–10	46 (36%)
	11–19	51 (40%)
	≥ 20	28 (22%)
	Not disclosed	2 (2%)
Ages of parents’ first child when introduced to GEP	0–10	33 (60%)
	11–19	21 (38%)
	≥ 20	1 (2%)

### Themes

#### Cultivating awareness of parents’ own schemas

One of the themes that emerged distinctly across all interviewees was the heightened sense of self-awareness about past issues and personal beliefs that were triggered when parenting their children. For instance, one parent noted that by getting her child to help out at home she had subconsciously placed too much expectation on the child and made her (the child) feel that she was not doing her chores well, as reflected in the following paragraph.

“When I had twins and was really busy, [my eldest daughter] would have been five or six. I was kind of like relying on her a bit more…to help me with [various] things. But I gained awareness of how this may be an issue when I learned GEP…I might have actually put too much on her for her age…made her feel like she has not done a good enough job…having reasonable limits was something very useful for me and [my eldest].”

(Interviewee 6)

This reflection, amongst many others, demonstrated how the principles of Good Enough Parenting can be used as a tool for reflexive parenting. This is shown in their daily parenting, by engaging in a self-critique, reconsidering their own actions in a timely manner, and making necessary adjustments. Another example is shown in one parent discussing how her personal “emotional inhibition” led her to primarily express concern about her children’s homework, instead of other aspects of their personal well-being. This was a theme that came up amongst several interviewees.

“I’m a caregiver, in terms of their physical needs…pocket money…meals…etc. But coming down to spending time with them one-to-one, [it is] a struggle for me. I am not really in touch with my feelings and it doesn’t quite help when my eldest girl is also very similar. But because of GEP, we try to find time one-on-one… [but] once I get it going, it actually it’s easier…getting to spend time, be able to make sure I have that connection, from there, she will open up, then it becomes more natural and become easier for both of us.”

(Interviewee 2)

Such reflexivity was also shown in greater awareness of coping styles. For example, avoidance was a common coping mechanism among some parents. One parent in particular cited that he would usually avoid engaging his children when they experienced emotions he felt unable to handle. Practicing Good Enough Parenting however, empowered and encouraged him to confront this avoidance tendency and stay engaged.

“When I see my children having emotions I can’t handle, my tendency would be to avoid it and just move on to do something else…this awareness help me not to give in and to be present and stay with my child [through] difficult incidents…by nature I don’t do that, so that really, and I see the positive outcome of that, because there is a genuine, real improvement in interactions with [my children].

(Interviewee 12)

#### Cultivating intentionality

One other theme that emerged from the interviews is how it encouraged parents to be more intentional in their actions and interactions. While this was a theme that appeared repeatedly for many interviewees, it was especially salient for working parents who have to shoulder multiple responsibilities, and even more so for single parents. For example, one single mother described how she learned to be more careful and encouraging with her choice of words, instead of reacting immediately when confronting her children.

“As a single mother, I work most of the time so I had very little interactions with them. But through the Repair and Reconnect I practiced through GEP…I learned how to connect with them… [during those times] I just felt that my choice of word is also very important, and I have to be more encouraging than telling them this this this this this you know what I mean? …I have to think about the right words to say, I think before I say, not just say whatever I want to say.”

(Interviewee 24)

Such intentionality shows up as a guiding principle when communicating with children, but also translates into the ways in which parents engage emotionally, as seen in the following examples.

“I think the thing that is hard to apply is being patient, not exasperating the child. Sometimes I feel that they are exasperating me so learning to be patient, and learning to not lose my cool, not fall into this what they called the vortex. Not fall into a certain vortex. And that is quite tough. Because sometimes even if I am very patient and I tried to be listener, it doesn’t mean they will talk more or they will even tell you things.”

(Interviewee 13)

“I think I definitely grew in patience, and I know that I am the adult over here, so I would have to learn to control my own emotions and triggers better in order to engage them.”

(Interviewee 54)

#### Working through developmental issues

One other theme discussed by interviewees is how the Good Enough Parenting approach helped them discover issues experienced by their children, and the roles they play in helping their children find success in their lives in their own ways. For example, one of the parents has a child who has a visual impairment. Practicing Good Enough Parenting enabled the parent to realize the significance of bonding time with his own child and through such time, helped the parent discover that his child had been very resilient.

“[My] boy was born with a squint…and at three years old, [developed] cross-eye. Only at six [years old] he went through the surgery to correct the squint. It meant that at home we need to be patching his eye, doing eye exercises with him for between 2–4 hours and that was ongoing right up to recently. So because of that I got to spend a lot of time with him. It reminded me [about] Connection and Acceptance. I need to accept him the way he is. It doesn’t matter if he is not reading, or not doing Math, or he doesn’t have nice hand writing. What was important to me was to have bonding time with him and that after his surgery, his life could be normal as possible. There are many disadvantages [he will face]. So I felt like I had [to be] patient with him. I struggled with practicing Healthy Autonomy with him as this boy can’t even see… But through my interactions with him I realized he actually [is] very bright…he picked up on all that spelling and everything on his own.”

(Interviewee 9)

Other developmental issues such as mental health development, Attention Deficit and Hyperactivity Disorder and global developmental delays were discussed by interviewees. They shared how Good Enough Parenting helped them to realize the emotional difficulties that their children were experiencing as a result of such challenges and how to use Good Enough Parenting strategies to connect with their children’s emotional needs and help them to overcome such issues.

“My [second child] was diagnosed with PTSD…she was in NUH for a few months and right now still going through therapy so she is coping…but at times there are triggers and she falls back into her symptoms… so it is hard and very challenging [to make connections]. But playfulness helps us to relax in intense situations…”

(Interviewee 18)

#### Responses to challenges at home

Participants of Good Enough Parenting also discussed different parenting challenges, especially in terms of communicating with their children at home. They felt that Good Enough Parenting, while not resolving all communication challenges at home, had served the function of raising awareness of various communication difficulties and issues between parents and their children. In doing so, Good Enough Parenting provided a way for them to begin settling misunderstandings and resolving past resentments. For example, one participant, who is a single mother, acknowledged her insecurity about depending on her children in her old age when she realized that her children were becoming more independent.

“[My children] are not so dependent on me [anymore], as they are [older] and very independent. I find myself more dependent on them…as a single mum the insecurity is there, you want somebody to look after you next time…I expect a lot from my children…You want to depend on your children, which I think I put a very heavy burden on them…[it’s] not only money, emotionally I also depend on them.”

(Interviewee 24)

Such insecurities and other issues were also better resolved through partnerships, using Good Enough Parenting as a mutual language for communication and conflict resolution. This was shown through a number of interviewees discussing how it was easier for them to communicate with their children who also understood the key principles of Good Enough Parenting.

“…it was difficult, but can be done. So I also thank my children because they also guide me along [as] they also know GEP. So sometimes they will send me things like ‘You can be more encouraging by saying this this this this this’…so they know what words are encouraging to them…and they will send me words.”

(Interviewee 24)

“Yes, [my children] also understand the part about parents as the base. Because it is about managing expectations, and obviously understanding all that repair and reconnect is helpful.”

(Interviewee 28)

“[In the past] I was punitive and if they cross the line they bear the consequences and sometimes I can get out of control I get very harsh in words and in actions … with GEP, I become less reactive and I [no longer] jump to conclusions easily…especially now when the child been exposed to GEP [they say] ‘Daddy can you cool down’ so they also learn to express [themselves] to avoid us getting into the vortex.”

(Interviewee 29)

#### Performing multiple roles

At home, parents play the roles of counsellor and educator, and Good Enough Parenting helps parents meet the emotional needs of connection and acceptance with their children. One parent does this by assessing her children’s strengths and weaknesses.

“I learned how to find out about my kids’ natural intelligences with [GEP]…So early on I understand that they’re all different. They took different pathways. And as long as this is what they are good at, this is what they enjoy doing…it works…but it’s not easy. Because kids have different temperaments and they have different challenges, so we must prepare to face those challenges.”

(Interviewee 51)

Some parents also play the role of the teacher at home, and shared their process of setting reasonable expectations with their children through Good Enough Parenting.

“I’m a school teacher and she had [many] problems in primary school…I would get very upset with her if she does not remember what I taught or if she does not understand simple things and I would also get upset when she cries. But after learning how to have reasonable expectations, and awareness of exasperating interactions, I learned to be patient with her. Before GEP, my expectation for her was of course to do well in school and all that. But after GEP, I try to focus more on what she might be actually good at doing. [My role] changed from helping her do well academically to helping her pursue whatever she feels she might be good at …to help her enjoy what she’s doing.”

(Interviewee 34)

“So one of [my children] generally doesn’t do very well [in exams] and that is a worry…but I know I have to be careful. The message that I want to put across is that yes I am concerned but our relationship is more important. In GEP we were also taught we need to focus on the efforts, focus on the process and not just the outcome.”

(Interviewee 13)

But this was not straightforward, with some interviewees discussing the tensions between being authoritative, being persuasive and being a listener. Such tensions are reflected in the following experience shared by one interviewee who discussed how he addressed the challenge of knowing when to be the final word on a discussion at home, and through a partnership with his spouse, learned to be more engaging and persuasive:

“I feel like I am in a position of authority especially earlier on in their lives. So my first responses is [usually] ‘I’m your dad, you do what I say.’ Another response that I usually have is if I feel that I am going to fight…I walk away to totally remove myself from the situation which is also not a healthy way…but one thing I learned through GEP is to be unified with my wife. So I guess to be united [means telling] the children ‘you know what, dad and mum have discussed, dad and mum know what’s best. There are already two wise heads into this I hope you guys are on board.’”

(Interviewee 14)

Such partnerships are not limited to spouses, with a number of interviewees discussing the importance of having a community in the form of church, friends and other parents learning and practicing Good Enough Parenting with them. Such reflections allude to the importance of being grounded in a learning community to make the practices of Good Enough Parenting deeper and more sustainable.

#### Learning process

Interviewees also shared how Good Enough Parenting has been instrumental in helping parents learn about the core emotional needs of their children, as well as their children’s temperaments and personalities. Interviewees with more than one child realized that each of their children behaved differently, so parents learned to utilize different approaches in communicating with them. In this process, they learned how to set their expectations differently for each of their children.

“[Physical discipline] did not work well with my youngest child at all…she would just be an emotional wreck, no matter how much we assure her…so we realize that she was sort of different from the first two…So [with my youngest], discipline is done before [she] sleeps…the energy is less so she is not so inclined to act out.”

(Interviewee 43)

“[Now my children] are older…and after hearing about how we should give them age appropriate tasks, to build that responsibility and autonomy. So recently, we got them to do chores so they could after dinner, they will take turns to mop floor or clean up the table, water the plants. They could do quite a number of things already. Fold their own clothes. They could bring their clothes to their room, keep their rooms neat and tidy…but [I] have to explain it to them. In the past if I would to do it, I would just command them to do it without really explaining the rationale behind it.”

(Interviewee 12)

Besides learning about their children, Good Enough Parenting also helped parents to learn about other parenting styles. For example, one parent reported that after he learned about autonomy granting and autonomy support, he became more supportive towards his children. Additionally, Good Enough Parenting also helped some of the parents know which core emotional needs of their children were lacking. One parent reported that when her daughter was hospitalized, instead of looking at the contents of her daughter’s phone, the parent decided to apply the Good Enough Parenting principles to practice acceptance and nurturance.

“Among the nurturing interactions [in GEP], they talk about autonomy granting and autonomy support. Okay so to me is to always be very supportive whatever you know, whatever decisions they make. Another thing I’ve learned as well is to practice empathy with my children…Be empathetic, put myself in their shoes, understand their world through their eyes and that helps a lot.”

(Interviewee 20)

“So we have this thing about parents meeting the core emotional needs of the children and also the fact the parents can have expectations on their kids. Okay, one very good example is [the issue of setting boundaries in terms of phone use]. We help our child to see that ‘hey your phone belongs to your mom because your mom is the one paying the subscription’ so in terms of training is like we help the child know that there are things that you think it’s your right and it’s your privilege but that it is your right but it is actually a privilege given to you…but [being mindful] of her emotional needs helped because I gave her more assurance that there is nothing she can do that can make me look down on her or reject her. So a lot of assurance and acceptance, and true enough after that she feels comfortable enough to tell me all that’s happening in her life and I think that openness at that critical junction lasted us until now. Today at 21 years old, we are like best buddies with each other. That’s how I feel about our relationship.”

(Interviewee 40)

This theme elucidates how Good Enough Parenting acts as a framework to incorporate other relevant knowledge and parenting strategies. It was also important to incorporate reflections in the learning of Good Enough Parenting itself, as shared by one interviewee:

“Yeah, I think it’s also very important to have this kind of interview, so that we can recount our good and bad experience, what is successful, what is not, and the journey still moving on, so is not that we have stop as a parent, yeah still a long way to improve, so I think it’s a good reminder for me to keep fighting on as a parent…it is a journey which is still ongoing.”

(Interviewee 17).

## Discussion

This article reports on a study into participant experience in using the Good Enough Parenting program [14]. Given the aim to provide insights into the experience of Good Enough Parenting amongst parents, this study utilized a qualitative approach involving 55 interviews with parents who have been practicing Good Enough Parenting for various periods of time. The ethnic distribution of the sample used was comparable to the national distribution of Singapore. All transcripts were extensively coded with good inter-coder reliability kappa value of 0.78 after two rounds of inter-coder discussions. Using CIT and thematic analysis the results provide evidence to support the overall effectiveness of Good Enough Parenting for both parents and their children, from the perspective of the parents.

At the thematic level, the findings support several of the theoretical aims of Good Enough Parenting. The program addresses negative early experiences that arise out of negative parent-child interactions (exasperation interactions) by helping parents to develop increased self-awareness of their own early maladaptive schemas and coping styles so that they could parent more reflexively. Parents reported enhanced patience and increased intentionality in their communications and actions which in turn reduced their reactionary and unhelpful responses to triggering interactions with their children. This is significant because reducing negative early experiences reduces the risk for poor outcomes for children [2, 3, 94, 95].

The program also aims to enhance mental well-being through its direct focus on positive parent-child interactions (nurturing interactions) and the enhancement of early adaptive schemas which in turn reduces risk factors for later mental health problems. Parents valued learning to identify and focus on their child’s unique strengths and characteristics, and learning to connect with their children emotionally to overcome challenges and foster resilience, particularly when faced with impairments and developmental disorders. As such they were better able to navigate the tensions between being emotionally supportive and limit-setting.

This inclusion of well-being and integrated focus on positive and negative characteristics and functioning in theory and intervention is seen as a key development within clinical psychology [96, 97] and psychiatry [98]. A child who develops early adaptive schemas will likely demonstrate self-directedness, self-reliance (amongst other things) which is in line with research showing that the use, over and above the possession, of strengths leads to enhanced well-being [99].

In line with behaviorally-based parenting programs, Good Enough Parenting provides concrete behavioral strategies and interventions for families that are explicitly linked to the nurturing and exasperation interactions, and therefore to the development of early adaptive schemas and reduction of early maladaptive schemas respectively [15, 16]. Parents appreciated these strategies when attempting to resolve misunderstandings and find ways to break out of repeated, habitual and negative patterns of interacting. They found that Good Enough Parenting provided a mutual language for both day-to-day communication and conflict resolution.

However, Good Enough Parenting goes beyond behaviorally-based parenting programs. It also focuses on the internal world of the child, specifically how children interpret interactions with their parents to form mental representations of themselves, the world and others, and how parents can either directly or indirectly provide learning opportunities for children that affect their emotional expression and their behavior. These are central ideas within any approach with cognitive-behavioral foundations such as schema therapy [100] and as such are important elements of an early intervention program. Above and beyond learning about different parenting approaches, parents valued learning about the constructs of core emotional needs and schemas, unique cornerstones of both schema therapy and Good Enough Parenting [17, 71].

Finally, the findings also show that the benefits are not limited to particular developmental phases for children; parents of children of all ages perceived Good Enough Parenting to be an effective parenting approach. This is significant because in some cultures, some parents continue to intensively parent, for example, through Over-Control even after their children have reached adulthood, not realizing that such practices will then become harmful [101]. Disciplining an adolescent may be constructive but repeating the same practice with adult children who have entered university, regardless of whether the adult has reached acceptable levels of socialization, could be harmful. At these later developmental stages societies cannot be called upon to provide unsolicited intervention in order to bring about the necessary changes to young adults who have not reached adequate socialization levels [73]. In these instances psychotherapy can serve an important role in compensating or filling in the gap for these unmet needs however effective parenting beginning in childhood will be a more effective and less costly means of helping children reach important socialization goals, become contributing members of society and lead more fulfilling lives [73].

### Limitations

The first limitation has to do with the relatively small number of interviewees and convenience sampling in most qualitative studies–although in the context of this study the number of interviewees is considered relatively large, the sample is limited because of its convenience. However, it could also be argued that the sample was very much a purposive one–given the focus on Good Enough Parenting, interviewees needed to be parents who practiced or have been exposed to Good Enough Parenting, even though some may not have practiced it for long. There could also be potential self-selection bias given that majority of the participants completed the full program, attended more than once since their first exposure to Good Enough Parenting, and participated in or were currently actively part of parent support groups practicing similar concepts. A sizeable group learned Good Enough Parenting when their children were between 0–10 years, and these children are now in adolescent or early adulthood years. This could have reinforced the key learnings over the years, compared to those who were exposed to the program only once. Given the qualitative nature of this research, it was not possible to establish the effects of attending the program more than once on outcomes.

Another limitation of qualitative studies is the relatively subjective nature of analysis as it can depend on the coders and how they interpret them. This was addressed somewhat through the inclusion of the inter-coder discussion and computation of inter-coder reliability, but due to the large number of interviewees it was not possible to compare the coding on all the transcripts. However, care was taken to ensure that coding began only when there was sufficient satisfaction that the coders had consistent and coherent understanding of the coding.

### Future research

Having established the effectiveness of the program from the participant perspective, the next step in overall intervention evaluation is to establish objective efficacy [75] for which RCTs are being planned. Current results will contribute to the design of the RCTs by informing the selection of patient-reported outcomes [102, 103].

## Conclusion

Regional authorities, educational services, police, medical, and social services are increasingly using or referring children and adolescents to early intervention programs. Such interventions commonly focus on either those children at high risk of developing adult psychological and behavioral problems (e.g., due to disadvantaged background or existing behavioral difficulties), or all within an area as part of population level investment in ensuring a psychologically healthy and productive next generation. Good Enough Parenting is designed to be used in both cases. Early intervention programs are effective [4] and cost saving versus the later financial impact of the psychopathology of the individuals who could have been helped by the program [5]. Schema therapy is a well-established cognitive behavioral approach with a firm evidence base for both theory and practice [104] with acceptability of specific techniques from the perspective of patients [105, 106] and therapists [107]. This large-scale qualitative study showed that Good Enough Parenting, the schema therapy early intervention and parenting program, has considerable acceptability to the end user participants. Further, the participants generally considered the program to be effective and their participation to be a positive experience.

## Supporting information

S1 Appendix Semi-structured interview using critical interview technique (Good Enough Parenting). (DOCX)
