# Supplementary material for: The Good Enough Parenting early intervention schema therapy based program: Participant experience
Source: PLoS One. 2021 Jan 22;16(1):e0243508. doi: 10.1371/journal.pone.0243508 (PMC7822299; doi:10.1371/journal.pone.0243508)
Supplement: S1 Appendix — (DOCX) [file pone.0243508.s001.docx]

The Good Enough Parenting Early Intervention Schema Therapy Based Program: Participant Experience

**S1 Appendix. Semi-structured interview using critical interview technique (Good Enough Parenting)**

Stage 1: Introduction

Hi, I am ____________________. I am here to have a chat about Good Enough Parenting and how it has been for you, including challenges encountered. This interview will take around 30-45 minutes, and thank you very much for your time and willingness to share your experiences with us. At any point, if there are questions that may make you uncomfortable, please know that you can let me know and skip over those questions. The data will be used only for analysis purposes, and we will not identify you in any of the data we may be using. Before we start, may I have your permission to record this interview? This is so that I can use this to make notes of our interview later.

Do you have any questions before we begin?

Thank you.

[Keywords exercise] Now, just to help us start the interview, I would like to invite you to do a reflection. In front of you is a blank sheet of paper: on one side, please write keywords that you would use to describe the kinds of interactions you had with your child/children before learning Good Enough Parenting, and on the other side, the kinds of interactions presently.

[Go through key Good Enough Parenting principles. Not necessary if interviewee is already familiar]

1. Could you tell me about yourself, and how many children you have? How old are your children, and how old were they when you started applying Good Enough Parenting in your parenting?
2. What were some of the most natural, and therefore, easiest principles to apply for you? Could you provide some examples?
3. What were some of the hardest principles to apply? Could you talk about them?

Stage 2: Identify critical incidents

1. Please take a moment to think about an incident that happened with your child that you felt went well. Tell me about what happened, the situation, who was involved? What did you or others involved do, or the factors that made you feel that the situation was positive?
2. What are some factors that contributed to the positivity of the incident? You are free to use what you’ve learned from Good Enough Parenting, but need not be limited your responses to them.
3. Now think of a time when things did not go very well and there was a conflict with your child. It could be a time when you were frustrated, even trying to apply Good Enough Parenting, and things just did not turn out as well as expected. Please tell me about what happened. What did you or others involved do, or the factors that made you feel that the situation did not turn out positively?
4. How did you resolve the situation?

Stage 3: Challenges, issues and emerging themes

1. Looking back, how do you think you have grown as a parent after learning Good Enough Parenting? [e.g. coping styles, meeting needs?]
2. How have your relationship with your child changed after learning Good Enough Parenting?
3. What do you think may be other factors that can impact your child’s development and also your own parenting? [e.g. marriage, spousal partnership, job, lifestyle?]
4. What do you think has been the biggest change in terms of your interactions with your child after learning Good Enough Parenting? [e.g. types of exasperating interactions which may have decreased]
5. What nurturing interactions would you like to see more of? What do you think are challenges that prevent them from happening more?
6. What exasperation interactions would you like to see less of? What do you think are reasons that trigger such interactions?
7. Do you have anything else you would like to add based on your own reflections of Good Enough Parenting?

Stage 4: Closing / Debrief

We have come to the end of the interview. I learned a lot from you today. For me, ____________________________________ were particularly insightful and made me think about _____________________________. Do you have anything to share about today’s interview? How was it for you, reflecting on the journey with Good Enough Parenting? Or further questions for me on the study?

We have come to the end of the interview. Thank you very much for your time!
